# Synergistic Antifungal Effect of Glabridin and Fluconazole

**DOI:** 10.1371/journal.pone.0103442

**Published:** 2014-07-24

**Authors:** Wei Liu, Li Ping Li, Jun Dong Zhang, Qun Li, Hui Shen, Si Min Chen, Li Juan He, Lan Yan, Guo Tong Xu, Mao Mao An, Yuan Ying Jiang

**Affiliations:** 1 Tongji University School of Medicine, Shanghai, China; 2 New Drug Research and Development Center, School of Pharmacy, Second Military Medical University, Shanghai, China; Louisiana State University, United States of America

## Abstract

The incidence of invasive fungal infections is increasing in recent years. The present study mainly investigated glabridin (Gla) alone and especially in combination with fluconazole (FLC) against *Cryptococcus neoformans* and *Candida* species (*Candida albicans*, *Candida tropicalis*, *Candida krusei*, *Candida parapsilosis* and *Candida Glabratas*) by different methods. The minimal inhibitory concentration (MIC) and the minimal fungicidal concentration (MFC) indicated that Gla possessed a broad-spectrum antifungal activity at relatively high concentrations. After combining with FLC, Gla exerted a potent synergistic effect against drug-resistant *C. albicans* and *C. tropicalis* at lower concentrations when interpreted by fractional inhibitory concentration index (FICI). Disk diffusion test and time-killing test confirming the synergistic fungicidal effect. Cell growth tests suggested that the synergistic effect of the two drugs depended more on the concentration of Gla. The cell envelop damage including a significant decrease of cell size and membrane permeability increasing were found after Gla treatment. Together, our results suggested that Gla possessed a synergistic effect with FLC and the cell envelope damage maybe contributed to the synergistic effect, which providing new information for developing novel antifungal agents.

## Introduction

Despite recent progress in the clinical management, invasive fungal infections are still a tricky problem and have a high mortality. *Candida* species are the fourth most important cause of hospital-acquired bloodstream infections. Besides, in developing countries systemic cryptoccocosis remains large and increasing [1]. The most common isolated *Candida* specie in clinical fungal invasive infection is *Candida albicans*, followed by *Candida tropicalis*, *Candida parapsilosis* and *Candida glabrata* [2]–[5]. *Cryptococcus neoformans* is the first or second most common cause of culture-proven meningitis [1]. One most common agent used in clinic is fluconazole (FLC). However, during long-time or repeated treatment, FLC resistance strains are easily developed [6]. The combination of two or more antifungal agents maybe a feasible policy to solve the problem.

Currently, researches on natural products which have potent synergisms with antifungal drugs have been raised. For example, retigeric acid B, a pentacyclic triterpenoid isolated from a lichen called *Lobaria kurokawae Yoshim*, can increase the susceptibilities of azole-resistant *C. albicans* strains in combination with azoles [7], [8]. Plagiochin E, a macrocyclic bis (bibenzyl) isolated from the liverwort *Marchantia polymorpha*, has antifungal activity and resistance reversal effects for *C. albicans* [9]. Besides, berberine chloride, baicalein, allicin, pure polyphenol curcumin I, pseudolaric acid B, eugenol and methyleugenol were also reported to have synergistic antifungal properties in combination with known antifungals [10]–[15].

Glabridin (Gla) [4-(8,8-dimethyl-2,3,4,8-tetrahydropyrano[2,3-f]chromen-3-yl)-benzene-1,3-diol] is a major active isoflavan isolated from *Glycyrrhiza glabra*. It has been reported that Gla had numerous beneficial properties, including antioxidant, anticancer, neuroprotective, anti-inflammatory activities, inhibiting fatigue or reversing learning and memory deficits in diabetic rats [16]–[23]. It possessed weak activity against *C. albicans*, *C. krusei*, *C. neoformans* and other filamentous fungi [24], [25]. However, to our knowledge, no study to date has focused on its interaction with FLC.

In this study, synergistic antifungal effect of Gla and FLC against FLC-resistant clinical isolates of *C. albicans* and other yeast fungi (i.e. *C. neoformans, C. tropicalis, C. parapsilosis, C. krusei* and *C. glabratas*) and the possible mechanisms were investigated.

## Materials and Methods

### Strains and chemicals

25 clinical isolates of FLC-resistant *C. albicans*, and one *C. neoformans* 32609, *C. tropicalis* 2718 and *C. parapsilosis* ATCC 22019 were kindly provided by the Changhai Hospital, Shanghai, China. *C. krusei* ATCC2340 and *C. glabrata* ATCC1182 were kindly provided by professor Changzhong Wang (School of integrated traditional and western medicine, Anhui university of traditional chinese medicine, Hefei, China). The susceptibilities of these strains to FLC were measured by broth microdilution method at advance. Frozen stocks of isolates were stored at −80°C in culture medium supplemented with 40% (vol/vol) glycerol and were subcultured twice at 35°C before each experiment. FLC (sigma Aldrich, St. Louis, MO) was obtained commercially. Gla (purity >98%) was obtained from Yuan Cheng Pharmaceutical Co. Ltd, China, and its initial stored concentration was 6.4 mg/ml in dimethyl sulfoxide (DMSO).

### Antifungal susceptibility testing

The minimal inhibitory concentrations (MIC) of Gla and FLC against the yeast strains were determined by broth microdilution method as described previously [10]. The yeast at final concentration of 10^3^ cells/ml in the RPMI 1640 liquid medium with serial (2×) dilutions of each drug were inoculated in 96-well flat-bottomed microtitration plates. After incubation at 35°C for 24 h or 72 h. Optical densities (OD) were measured at 630 nm with a microtitre plate reader (Thermolabsystems Multiskan MK3), and background optical densities were subtracted from that of each well. MIC_80_ was determined as the lowest concentration of the drugs that inhibited growth by 80% compared with that of drug-free wells. DMSO comprised <1% of the total test volume. The quality control strain, *C. parapsilosis* ATCC 22019 was included in each susceptibility test to ensure quality control. The MIC range of FLC to *C. parapsilosis* ATCC 22019 was from 0.5 µg/ml to 4 µg/ml, which meant this test was acceptable.

### Checkerboard microdilution assay

Assays were performed on all isolates according to broth microdilution checkerboard method [10]. The initial concentration of fungal suspension in RPMI 1640 medium was 10^3^ cells/ml, and the final combination concentrations ranged from 0.125 to 64 µg/ml for FLC and 1 to 16 µg/ml for Gla. The final concentration for FLC or Gla alone ranged from 0.125 to 64 µg/ml. 96-well flat-bottomed microtitration plates were incubated at 35°C for 24 h or 72 h. OD was measured at 630 nm, MIC was determined as the above.

The data obtained by the checkerboard microdilution assays were analyzed using the model-fractional inhibitory concentration index (FICI) method based on the Loewe additivity theory. FICI was calculated by the following equation: FICI = FIC A+FIC B, where FIC A is the MIC of the combination/the MIC of drug A alone, and FIC B is the MIC of the combination/the MIC of drug B alone. Among all of the FICIs calculated for each data set, the FICImin was reported as the FICI in all cases unless the FICImax was greater than four, in which case the FICImax was reported as the FICI. Synergy was defined as an FICI value of ≤0.5, while antagonism was defined as an FICI value of >4, addition was defined as an FICI value of 0.5< FICI≤1. An FICI result between 1 and 4 (1< FICI≤4) was considered indifferent [7]. The fractional fungicidal concentration index (FFCI) was calculated the same.

### Agar disk diffusion test

*C. albicans* 103 (one FLC-resistant isolate with a MIC of 32 µg/ml for Gla) and other yeast strains were tested by agar diffusion test [10]. 3 ml of aliquot of 10^6^ cells/ml suspension was spread uniformly onto the yeast peptone dextrose (YPD) agar plate with or without 64 µg/ml FLC. Then, 6 mm paper disks impregnated with Gla alone or in combination with FLC were placed onto the agar surface. There was 5 µl DMSO in control disks. Photos were taken after incubation at 35°C for 48 h.

### Time-killing test

*C. albicans* 103 and other yeast strains were prepared at the starting inoculum of 10^5^ cells/ml. The concentrations were 4, 8, 16 µg/ml for Gla and 8 µg/ml for FLC, DMSO comprised <1% of the total test volume. At predetermined time points (0, 4, 8, 12, 16 and 24 h) after incubation with agitation at 35°C, a 100 µl aliquot was removed from every solution and serially diluted 10-fold in sterile water. A 100 µl aliquot from each dilution was spread on the sabouraud dextrose agar plate. Colony counts were determined after incubation at 35°C for 48 h. Fungicidal activity was defined as a ≥3 log_10_ reduction from the starting inoculum. Synergism and antagonism were defined as a respective decrease or increase of ≥2 log_10_ CFU/ml in antifungal activity produced by the combination compared with that by the more active agent alone [26].

### Cell growth test

*C. albicans* 103 was prepared at the starting inoculum of 10^6^ cells/ml in glass tubes. Different concentrations of Gla (2, 4, 8, 16 µg/ml) and FLC (2, 4, 8, 16, 32, 64 µg/ml) alone or the combinations of Gla (2, 4, 8, 16 µg/ml) and FLC (2, 4, 8, 16, 32, 64 µg/ml) were added into tubes. After incubation with agitation at 35°C for 24 h, pictures were taken. Aliquot was removed from each tube and serially diluted 10-fold in sterile water. A 100 µl aliquot from each dilution was spread on the sabouraud dextrose agar plate. Colony counts were determined after incubation at 35°C for 48 h.

### Cell membrane permeability

Membrane permeabilization of *C. albicans* was detected according to a previous study [27]. Briefly, *C. albicans* 103 (1×10^7^ cells/ml) were incubated with 10 µM calcein acetoxymethyl ester (Fanbo biochemicals, China) for 2 h, The cells were then washed three times and *C. albicans* (1×10^7^ cells/ml) was transferred to tubes. After treatment with or without FLC (64 µg/ml), MCZ (64 µg/ml) and Gla (16 µg/ml, 32 µg/ml, 64 µg/ml) for 3 h. The cells were washed three times and about 10,000 cells were acquired for flow cytometry analysis (Maflo Astrios flow cytometer). Experiments were repeated at least two times independently on separate days.

### Cell wall inhibitors sensitivity test

Congored and calcofluorwhite (CFW) were incorporated into YPD agar plates at 100 µg/ml and 15 µg/ml, respectively. Yeast cells were grown in YPD medium with or without Gla (8, 16 µg/ml) for 12 h and 3 µl drops of serially diluted suspensions were inoculated into plates. After incubation for 48 h at 30°C, pictures were taken.

## Results

### The combination of Gla and FLC against clinical FLC-resistant *C. albicans*

The MIC values of Gla tested alone or in combination with FLC in FLC-resistant *C. albicans* were shown in Table 1. According to the interpretive breakpoints for FLC (<8 µg/ml and ≥64 µg/ml, respectively), 25 clinical FLC-resistant *C. albicans* were selected. The MICs of Gla against all tested strains ranged from 32 µg/ml to 64 µg/ml. When MIC-like assays were performed for FLC in the presence of fixed subinhibitory concentrations of Gla (4 µg/ml), the median MICs of FLC decreased from 128 µg/ml to 1 µg/ml in resistant strains (32-fold to 512-fold reductions). According to the analysis of FICI method, synergism was observed in all 25 tested strains (FICIs<0.2).

**Table 1:** MICs and MFCs of Gla alone and in combination with FLC against 25 clinical FLC-resistant *C. albicans.*

	MIC (μg/ml)		MFC (μg/ml)	
	median	range	median	range
FLC	128	64–>256	>256	>256
Gla	32	32–64	64	32–64
FLC/Gla^a^	1/4	1–1/4–4	8/16	4–16/8–16
FIC index	0.13	0.04–0.14	0.27	0.26–0.31
Interaction effect (n/%)^b^	Syn (25/100)		Syn (25/100)	

a MIC and MFC in combination expressed as [FLC]/[Gla].

b Syn, synergism. The number of strains and percentage for the interaction effect were shown.

### The combination of Gla and FLC against other varied FLC-susceptibility strains

We also tested antifungal effects of Gla alone or in combination with FLC in FLC-sensitive *C. albicans* and the other yeast strains (*C. neoformans, C. tropicalis*, *C. krusei*, *C. parapsilosis* and *C. glabratas*) (Table 2). In these strains, the range of MICs of Gla tested alone was from 16 µg/ml to 64 µg/ml, when in combination with FLC the MICs of Gla ranged from 1 µg/ml to16 µg/ml. In *C. tropicalis* and *C. krusei*, the MIC of FLC were reduced from >64 µg/ml to 4 µg/ml or 8 µg/ml respectively after in combination with Gla. In *C. glabratas*, no synergistic effect was observed.

**Table 2:** MICs and MFCs of Gla alone and in combination with FLC against varied yeast strains.

Yeast strains	MIC (µg/m			FICI	MFC (µg/m			FFCI
	FLC	Gla	FLC/Gla		FLC	Gla	FLC/Gla	
*C. albicans* SC5314	1	32	1/1	1.03	>64	32	16/16	0.63
*C. tropicalis* 2718	>64	64	4/8	0.16	>64	64	16/16	0.38
*C. neoformans* 32609	2	16	≤0.125/8	0.56	>64	32	8/16	0.56
*C. parapsilosis* ATCC22019	2	64	2/1	1.02	>64	64	32/16	0.50
*C. krusei* ATCC2340	>64	64	8/16	0.31	>64	64	>64/16	1.25
*C. glabrata* ATCC1182	>64	64	64/16	0.75	>64	64	>64/16	1.25

### In combination with FLC, Gla at lower concentrations exhibits fungicidal effect for FLC-resistant *C. albicans* by different methods

Cells from the microdilution assays after incubation with Gla, FLC or the combination of Gla and FLC at various concentrations were plated on the sabouraud dextrose agar plate to count the colony forming unit (CFU) for determination of the MFC_100_ (the minimal concentration with complete cell killing, i.e. no CFU counted). As shown in Table 1, the MFC of FLC can be much higher than the MIC and complete cell killing was not achievable. The range of the MFC of Gla was from 32 µg/ml to 64 µg/ml. When in combination with FLC (4 µg/ml or 8 µg/ml), Gla at 16 µg/ml showed fungicidal effect against all strains tested.

**Figure 1:**
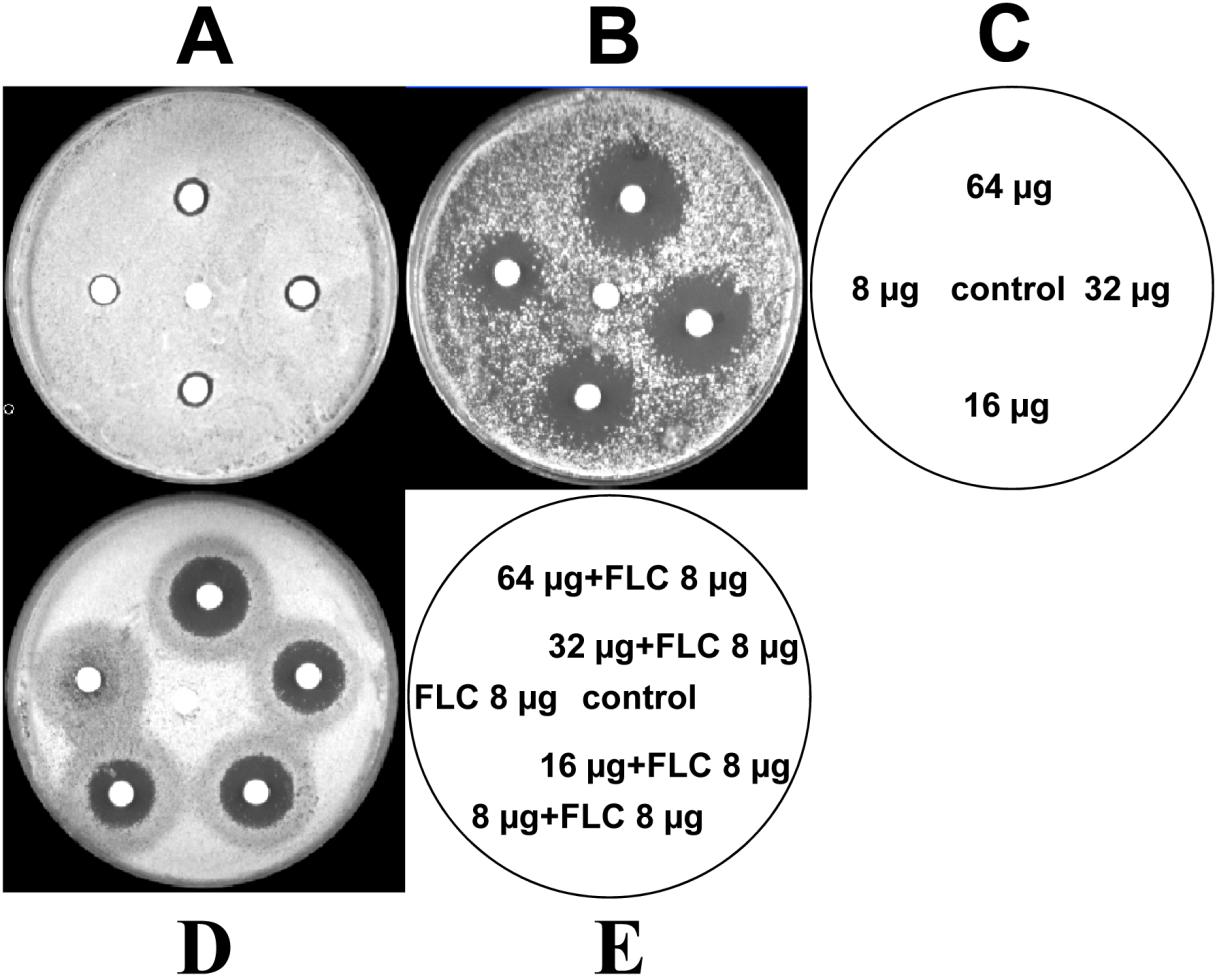
Agar disk diffusion assay of different concentrations of Gla combined with FLC against *C. albicans* 103. Panels A and D show agar plates, and panel B shows an agar plate containing 64 µg/ml FLC. Panel C describes the images for panels A and B, which containing 64, 32, 16, 8 µg of Gla or DMSO as control per disc. Panel E describes the image for panel D, the combination of Gla (64, 32, 16, 8 µg) with FLC (8 µg) or FLC (8 µg) alone or just DMSO as control were contained in each disc.

Further to visualize their synergistic fungicidal effect, different concentrations of Gla and the combination with FLC (8 µg/ml) were analyzed by agar disk diffusion assay. Gla alone at 64, 32, 16, 8 µg per disc had minimal fungicidal activity against the FLC-resistant *C. albicans* 103. While FLC at 8 µg per disc showed weak inhibition effect against *C. albicans*, the halo surrounding the disc was cloudy with colony (Fig. 1A). Interestingly, when FLC was combined with Gla, the halo surrounding the disc was significantly clearer. The diameters of the zones were clearer and larger than those of either drug alone on the plain agar plate, which was an indication of potent synergistic fungicidal activity (Fig. 1D). Similarly, on the agar plate (containing 64 µg/ml FLC), Gla also yielded significantly clearer and larger zones at 64, 32, 16, 8 µg per disc (Fig. 1B).

**Figure 2:**
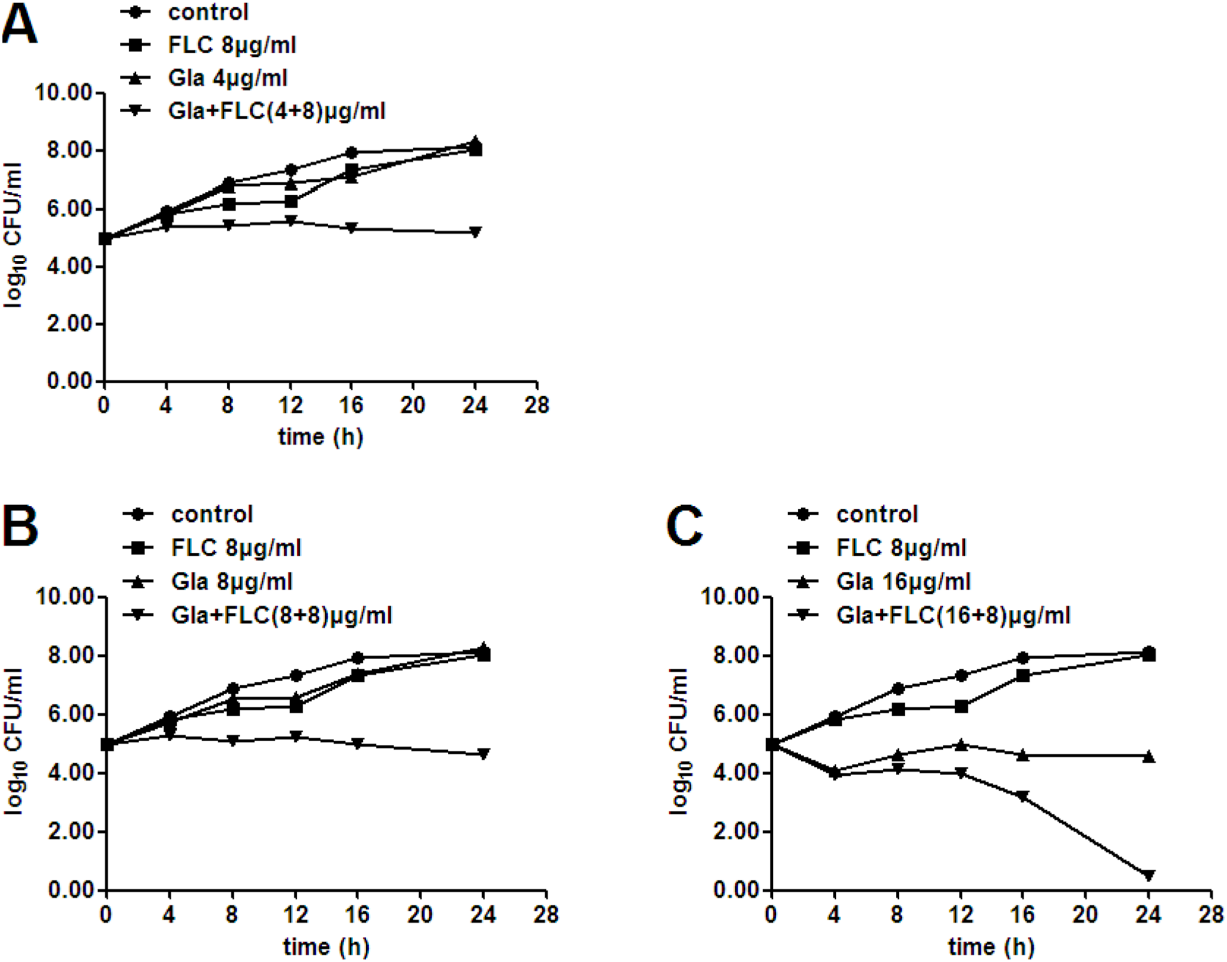
Time killing curves of *C. albicans* 103 treated with different concentrations of Gla and FLC. FLC-resistant *C. albicans* 103 were treated with FLC (8 µg/ml), Gla (4 µg/ml) and FLC+Gla (4+8) µg/ml (A), Gla (8 µg/ml) and FLC+Gla (8+8) µg/ml (B) or Gla (16 µg/ml) and FLC+Gla (8+16) µg/ml (C) for 24 h. Aliquots were obtained at the indicated time points and serially dilutions were spreaded on agar plates. Colony counts were determined after 48 h incubation.

In addition, their synergistic fungicidal effect was confirmed by time-killing test (Fig. 2). Gla alone at 16 µg/ml showed fungicidal effect and led to a decrease of 3.57–log_10_ CFU/ml at 24 h. No appreciable antifungal activity of FLC alone at 8 µg/ml was observed, but the combination of FLC (8 µg/ml) and Gla (4, 8 or 16 µg/ml) yielded 3.14, 3.62 or 4.10–log_10_ CFU/ml reductions compared with Gla alone at 24 h (Table 3). Besides, the combination of Gla at 16 µg/ml and FLC at 8 µg/ml almost resulted in a complete cell-killing at 24 h (Fig. 2C).

**Table 3:** Decrease in log_10_ CFU/ml of yeast strains using different concentrations of Gla combining with FLC at 24 h.

FLC+Gla (µg/ml)	Mean (±SD) decrease in log_10_ CFU/ml compared with Gla alone			
	*C. albicans* 103	*C. albicans* SC5314	*C. tropicalis* 2718	*C. neoformans* 32609
8+4	3.14 (0.08)			
8+8	3.62 (0.11)	1.51 (0.08)	1.64 (0.09)	4.42 (0.12)
8+16	4.10 (0.30)	1.82 (0.09)	3.16 (0.11)	4.50 (0.11)

**Figure 3:**
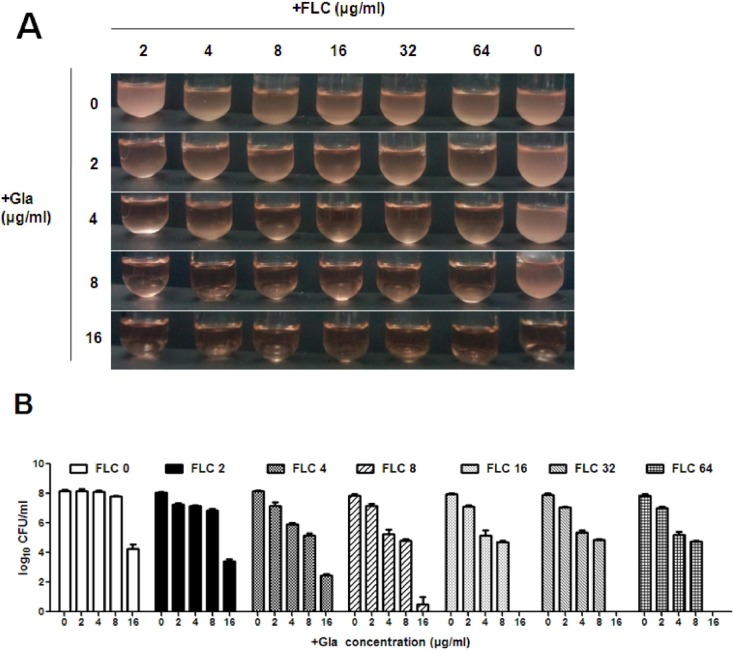
Growth condition of *C. albicans* 103 treated with different concentrations of Gla and FLC. Exponetially growing FLC-resistant *C. albicans* 103 were treated with or without different concentrations of Gla (2, 4, 8, 16 µg/ml) and FLC (2, 4, 8, 16, 32, 64 µg/ml) alone or the combinations of Gla and FLC in a shaking incubator. (A) Pictures of the growth condition of *C. albicans* were taken after 24 h incubation. (B) Aliquots from each tube were obtained at 24 h and serially dilutions were spread on agar plates. The number of *C. albicans* in each tube was determined by counting colonies after 48 h incubation.

In order to determine the relationship between the synergistic effect and the dosage of Gla and FLC, different concentrations of Gla (2 µg/ml–16 µg/ml) and FLC (2 µg/ml–64 µg/ml) were used in the cell growth test. Our results indicated that the synergism of the two drugs depended more on the concentration of Gla than FLC (Fig. 3). 4 µg/ml and 8 µg/ml Gla alone had no antifungal effect, while 64 µg/ml FLC had a weak antifungal activity. The antifungal effect was improved significantly after the two drugs used together at different concentrations except when FLC used at the concentration of 2 µg/ml. More specifically, 16 µg/ml Gla alone had an antifungal effect, while after combining with FLC (4 µg/ml–64 µg/ml), significantly synergistic effects were observed, and even complete cell killing activities were found when the concentration of FLC were above 16 µg/ml. Interestingly, the synergistic effects of the two drugs were unchanged when the dose of FLC declined from 64 µg/ml to 16 µg/ml, but when the combination concentration of FLC was below 16 µg/ml, the synergistic effect was lessened with the doseage of FLC decreasing.

### Synergistic effect of FLC and Gla against other yeast strains

**Figure 4:**
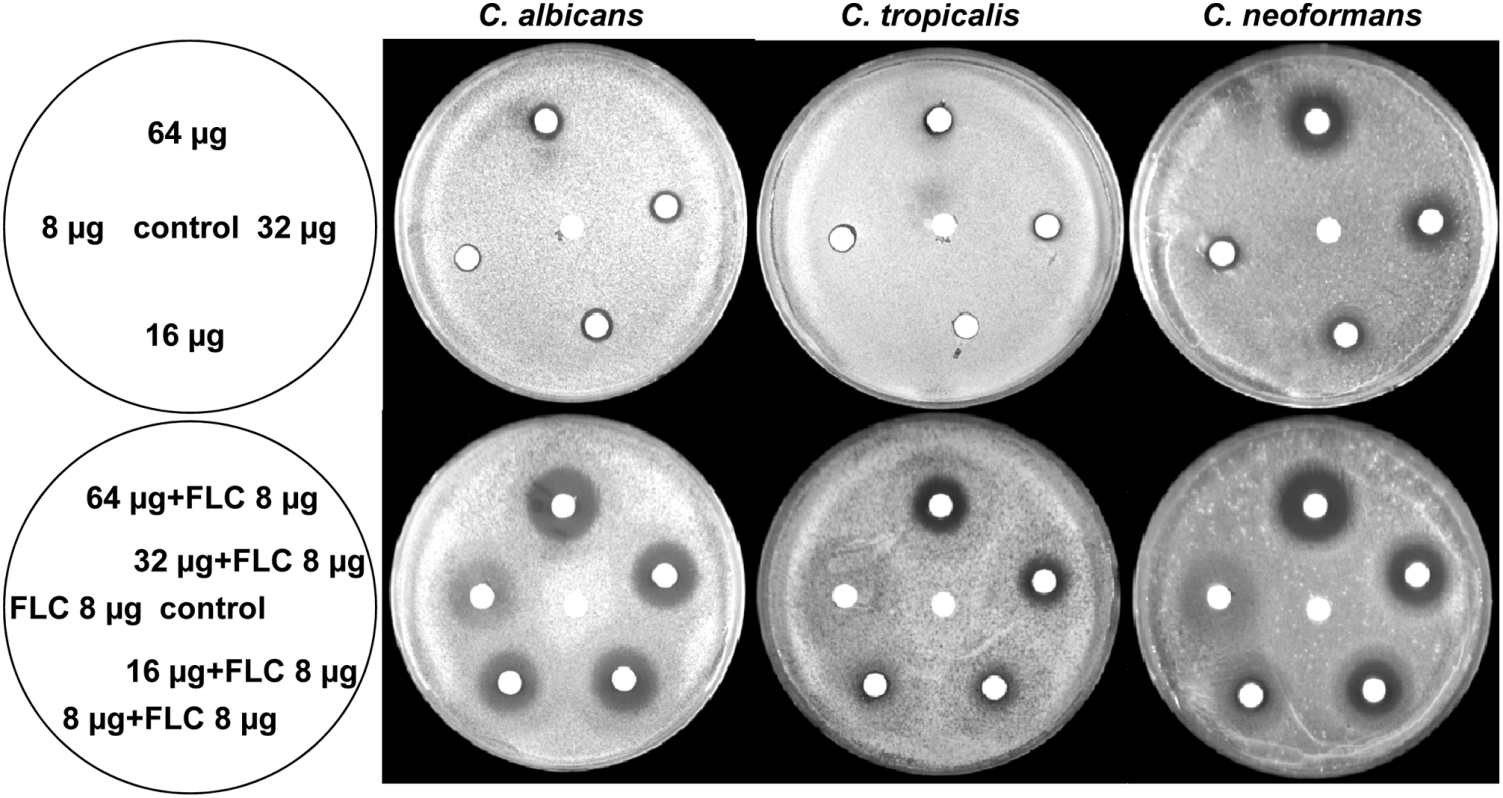
Agar disk diffusion assay of Gla alone or in combination with FLC against *C. albicans* SC5314, *C. tropicalis* and *C. neoformans*. Upper agar plates of disks were impregnated with 64, 32, 16 and 8 µg of Gla or 5 µl of DMSO as control disk. In lower agar plates, disks were impregnated with FLC+Gla (64+8) µg, FLC+Gla (32+8) µg, FLC+Gla (16+8) µg, FLC+Gla (8+8) µg, FLC (8 µg) or 5 µl of DMSO as control disk. Left sketch panels describe the images for the right agar plates.

The interactions of FLC and Gla against the other yeast strains (i.e. FLC-senstive *C. albicans*, *C. tropicalis, C. neoformans*, *C. parapsilosis, C. krusei* and *C. glabrata*) were investigated by MFCs, agar disk diffusion assay and time-killing test. As shown in Table 2, the strains showed varied susceptibility to Gla and FLC, and there was no apparent correlation between the susceptibility towards Gla and the susceptibility towards FLC. Consistent with results from the disc diffusion assays, the *C. krusei* and *C. glabrata* were highly resistant to FLC. The range of MFC of Gla for each strain tested was from 32 µg/ml to 64 µg/ml, supporting its fungicidal property. Synergistic fungicidal interactions between Gla and FLC were also observed in *C. tropicalis* by counting cells from the microdilution assay. The halo surrounding the discs with FLC and Gla was significantly clearer (Fig. 4) and the diameters of the zones were larger than those of either drug alone on the plain agar plate for FLC-senstive *C. albicans*, *C. tropicalis* and *C. neoformans*. Besides, the FLC+Gla combination yielded a decreased CFU compared with Gla alone in FLC-senstive *C. albicans*, *C. tropicalis* and *C. neoformans*, and even greater reductions were observed in *C. neoformans* and *C. tropicalis* (>3 Log_10_ CFU/ml decrease, fungicidal effect can be defined) (Table 3). However, the synergistic fungicidal effect of FLC and Gla was not observed in *C. krusei* and *C. glabrata* by agar disk diffusion assay and time-killing test (results not shown), which was consistent with FFCI values of the previous tests.

### Effect of Gla on the cell envelope

**Figure 5:**
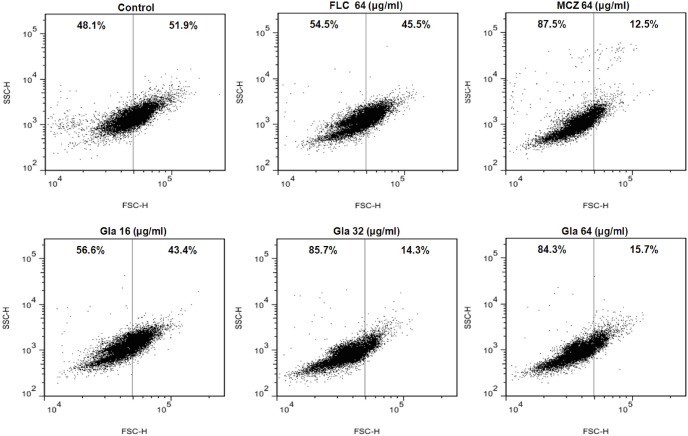
Changes in cell size (forward scatter-side scatter) in the presence of Gla. FLC-resistant *C. albicans* 103 were treated with or without Gla (16, 32, 64 µg/ml), FLC (64 µg/ml), MCZ (64 µg/ml) for 3 h. Then the cells were analyzed by flow cytometry.

Flow cytometry analysis (side scatter [SSC]-forward light scatter [FSC]) showed that *C. albicans* 103 treated with FLC underwent weak cell shrinkage, while the cells treated with MCZ exhibited a significant decrease in cell size, as evidenced by the decrease in forward light scattering. Interestingly, changes in cell size were also observed after exposure to Gla, especially to Gla at the concentration of 32 µg/ml and 64 µg/ml (Fig. 5).

**Figure 6:**
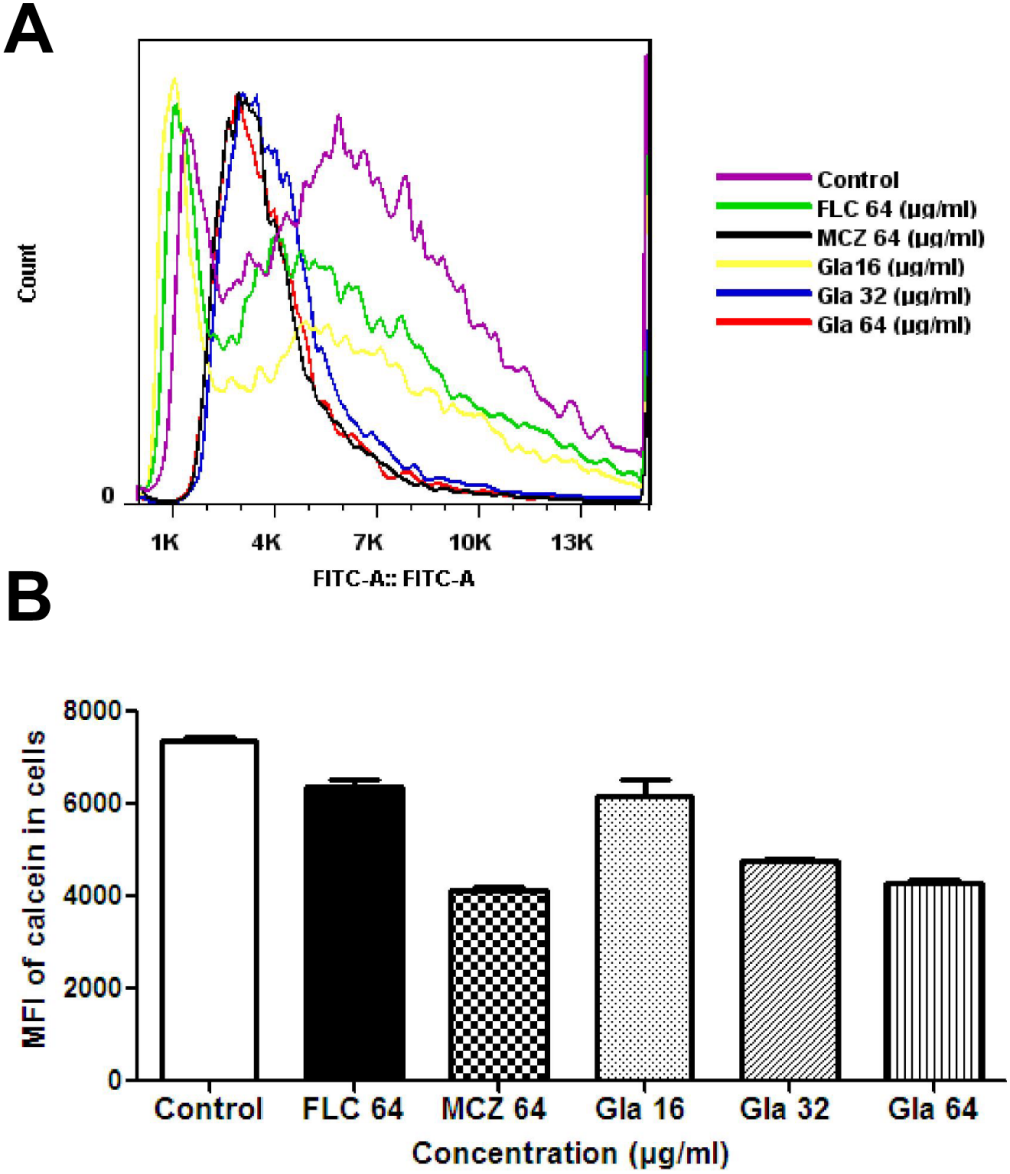
The influence of Gla on cell membrane permeability. *Candida* cells (1×10^7^ cells/mL) were cultured in the presence of 10 µM calcein acetoxymethylester for 2 h. After washing four times, the cells were cultured in the presence or absence of Gla (16, 32, 64 µg/ml), FLC (64 µg/ml), MCZ (64 µg/ml) for 3 h. Cellular fluorescence intensities of calcein in the cells were analyzed by flow Cytometry.

Calcein AM is a non-fluorescent derivative of calcein that can readily diffuse across membranes. Once within the cytoplasms of target cells, calcein AM is hydrolyzed by cytoplasmic esterases, yielding membrane-impermeable calcein which could be loaded into intact cells. After incubation with calcein AM, the cellular fluorescence of calcein was detected and quantified by flow cytometry to evaluate the effect of Gla on the cell membrane permeabilization. Results showed that cellular calcein was markedly decreased by treatment of *Candida* cells with different concentrations of Gla (16 µg/ml, 32 µg/ml, 64 µg/ml), while by FLC a slight reduction of cellular calcein was observed (Fig. 6).

**Figure 7:**
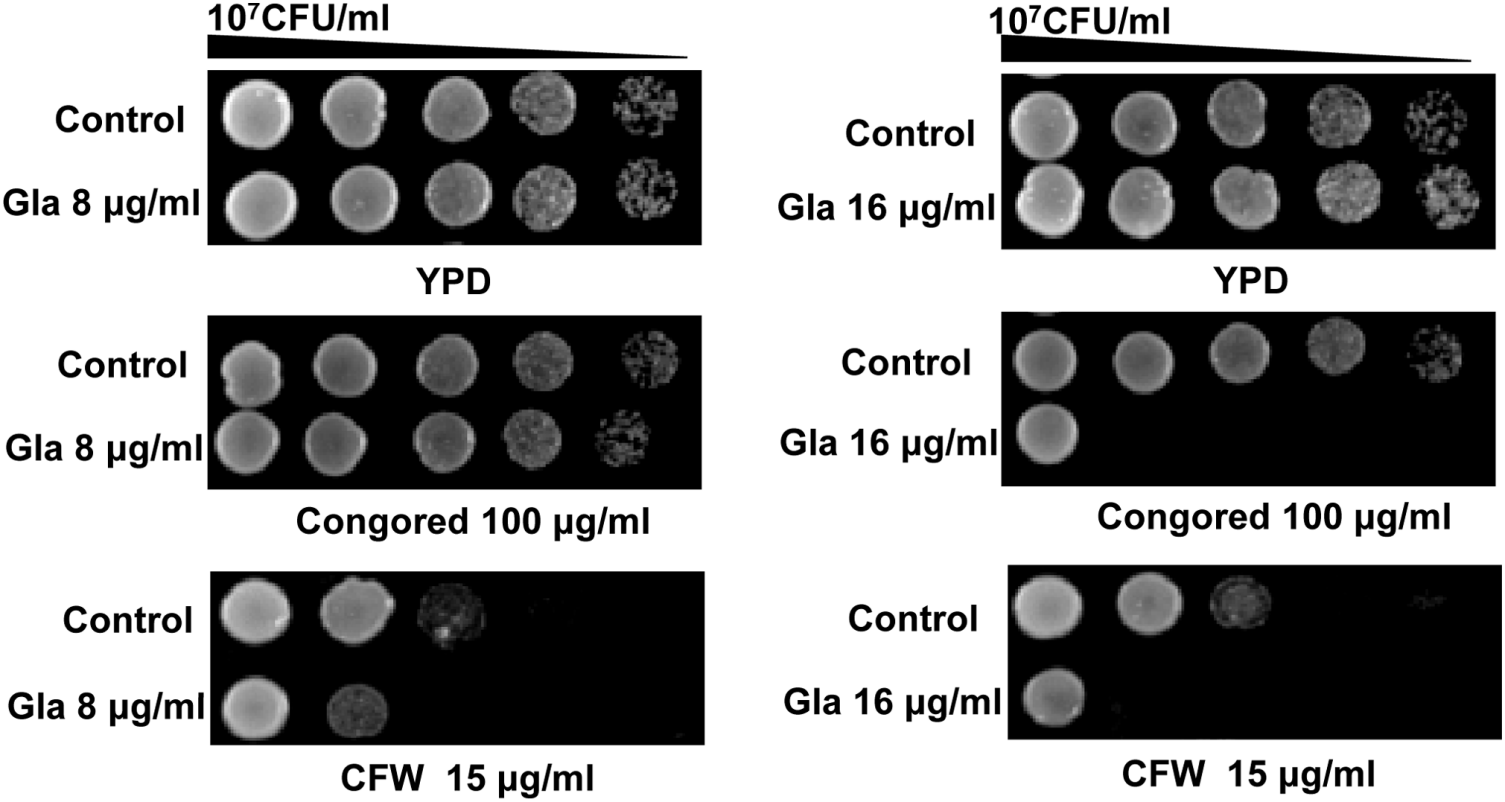
Susceptibilities of *C. albicans* 103 to cell wall inhibitors after treated with or without Gla. Exponetially growing FLC-resistant *C. albicans* 103 were treated with or without Gla (8, 16 µg/ml) for 12 h in YPD. Then they were diluted to 1×10^7^ CFU/ml and 3 µl from 1∶5 serial dilutions were spotted onto YPD agar plates containing 15 µg/ml CFW or 100 µg/ml congored.

We also investigated the effect of Gla on the cell wall carbohydrates. Spot assays indicated that 16 µg/ml Gla treatment made *C. albicans* become more sensitive to both cell wall inhibitors (CFW and congored) compared with the control cells (Fig. 7). We used concanavalin A, calcofluorwhite (CFW) and specific anti-β-glucan primary antibody to stain the carbohydrates (mannan, chitin and glucan). However, fluorescence micrographs did not show obvious change in the three cell wall layers (Fig. S1).

## Discussions

A large number of natural products from plants are reported to possess potent antifungal properties in recent years, such as terpene derivatives, flavans, nucleosides, peptides, alkaloids, saponins and sterols [28]. Gla is an isoflavan from *G. glabra* root. Previous studies have been reported that the alcohol or ethanol extract of the roots of *G. glabra* possessed weak antifungal activity against *C. albicans*, *Arthrinium sacchari*, *Chaetonmium funicola* and various filamentous fungi [24], [29], [30]. Synergistic activity of Gla and nystatin against oral *C. albicans* has been demonstrated previously [25]. But to our knowledge, there was no investigation on the combination of Gla and FLC against fungi.

Here, we demonstrated that Gla had weak antifungal activity against different fungi such as *Candida species and C. neoformans,* consisting with the previous reports [24], [25],[29],[30]. When combining with FLC, Gla exerted a strong synergistic antifungal effects at lower concentrations. Different assays indicated a potent synergistic effect of Gla and FLC against FLC-resistant *C. albicans*, *C. neoformans* and *C. tropicalis*. The synergistic effects depended more on the concentration of Gla. According to Celine Messier’s study on the toxic effect of Gla for oral epithelial cells (67% cell viability at 10 µg/ml), the concentration of Gla required for reducing the MIC of FLC ranged from 1 µg/ml to 4 µg/ml, below the concentration which significantly reduced cell viability [25]. In order to determine whether this property of glabridin is specific to other isoflavans, we selected a second isoflavan equol and tested the interaction of equol and FLC against FLC-resistant *C. albicans* by checkerboard microdilution assay. The results showed a weak synergistic effect between equol and FLC. The MIC of equol alone was >1280 µg/ml, only when the concentration of equol was at 32 µg/ml a synergism was observed between equol and FLC, the concentration of FLC was reduced from >64 µg/ml to 4 µg/ml. This may suggested that there were synergistic effects between isoflavans and FLC, but the antifungal activities and synergisms of isoflavans and FLC were different for their distinct chemical structures.

The synergistic antifungal effect of Gla has not been characterized. Similar studies on the mode of synergism as follows: increasing reactive oxygen species (ROS) to accelerate apoptosis [31], inhibiting drug efflux pumps to increase intracellular drug concentration [11], [32], targeting the ergosterol biosynthesis pathway to increase the fluidity for the resulted ergosterol depletion [32]. We tested the membrane sterols of *C. albicans* after treated with Gla alone or in combination with FLC by GC/MS, but none obvious sterols change was observed in Gla treatment cells (data not shown). This suggested that the synergism of Gla and FLC may not be related to the inhibition of membrane sterols synthesis. Previous study have displayed that isoflavan equol was capable of changing *Candida* cell membrane integrity by formation of membrane lesions and cell surface abnormalities against *C. albicans* [33]. Besides, flavans catechin hydrate and epigallocatechin gallate were also identified to have synergistic effect with FLC against *C. tropicalis*, cell shrinkage and plasma membrane damage were observed in the combination [34]. In our study, similar cell envelope changes were found in the *Candida* cells treated with Gla. A significant decrease in cell size and an increase of cell membrane permeability were observed in *C. albicans* after the treatment with Gla. However, *Candida* cells treated with Gla became more sensitive to cell wall inhibitors. We further stained the carbohydrate of the cell wall (mannan, chitin and glucan), while no obvious change of cell wall after Gla treatment was observed (Fig. S1).

In conclusion, the present study first demonstrated that Gla could enhance the antifungal effect of FLC, especially showed strong synergistic effect against *C. albicans, C. neoformans* and *C. tropicalis.* Their synergism maybe related to the effect of Gla on the cell envelope. Gla may serve as a pro-natural product for fungal infection treatment. Further studies should be carried out to identify its relationship of activity and structures.

## Supporting Information

Figure S1 **Fluorescence micrographs of the cell wall structures of** ***Candida albicans*** **by the treatment of Gla.** Exponentially growing cells treated with or without 32 µg/ml Gla were stained by 50 µg/ml Concanavalin A alexa fluor 488 conjugate for mannan, 30 µg/ml Calcofluorwhite for chitin, or specific anti-β-glucan primary antibody and Cy3-labeled goat-anti mouse secondary antibody for glucan. Then cells were scanned under a Leica confocal laser scanning microscope and micrographs were acquired.
(TIF)

## References

[1] Pukkila-Worley R, Mylonakis E (2008) Epidemiology and management of cryptococcal meningitis: developments and challenges. Expert Opin Pharmacother 9: 551–560. 18312157 10.1517/14656566.9.4.551

[2] Warnock DW (2007) Trends in the epidemiology of invasive fungal infections. Nihon Ishinkin Gakkai Zasshi 48: 1–12. 17287717 10.3314/jjmm.48.1

[3] Morace G, Borghi E (2010) Fungal infections in ICU patients: epidemiology and the role of diagnostics. Minerva Anestesiol 76: 950–956. 21102391

[4] Yapar N, Pullukcu H, Avkan-Oguz V, Sayin-Kutlu S, Ertugrul B, et al (2011) Evaluation of species distribution and risk factors of candidemia: a multicenter case-control study. Med Mycol 49: 26–31. 20662635 10.3109/13693786.2010.501344

[5] Zirkel J, Klinker H, Kuhn A, Abele-Horn M, Tappe D, et al (2012) Epidemiology of *Candida* blood stream infections in patients with hematological malignancies or solid tumors. Med Mycol 50: 50–55. 21696259 10.3109/13693786.2011.587211

[6] Horn DL, Neofytos D, Anaissie EJ, Fishman JA, Steinbach WJ, et al (2009) Epidemiology and outcomes of candidemia in 2019 patients: data from the prospective antifungal therapy alliance registry. Clin Infect Dis 48: 1695–1703. 19441981 10.1086/599039

[7] Sun L, Sun S, Cheng A, Wu X, Zhang Y, et al (2009) In vitro activities of retigeric acid B alone and in combination with azole antifungal agents against *Candida albicans* . Antimicrob Agents Chemother 53: 1586–1591. 19171796 10.1128/AAC.00940-08 PMC2663064

[8] Chang W, Li Y, Zhang L, Cheng A, Liu Y, et al (2012) Retigeric acid B enhances the efficacy of azoles combating the virulence and biofilm formation of *Candida albicans* . Biol Pharm Bull 35: 1794–1801. 22863995 10.1248/bpb.b12-00511

[9] Guo XL, Leng P, Yang Y, Yu LG, Lou HX (2008) Plagiochin E, a botanic-derived phenolic compound, reverses fungal resistance to fluconazole relating to the efflux pump. J Appl Microbiol 104: 831–838. 18194250 10.1111/j.1365-2672.2007.03617.x

[10] Quan H, Cao YY, Xu Z, Zhao JX, Gao PH, et al (2006) Potent in vitro synergism of fluconazole and berberine chloride against clinical isolates of *Candida albicans* resistant to fluconazole. Antimicrob Agents Chemother 50: 1096–1099. 16495278 10.1128/AAC.50.3.1096-1099.2006 PMC1426442

[11] Huang S, Cao YY, Dai BD, Sun XR, Zhu ZY, et al (2008) In vitro synergism of fluconazole and baicalein against clinical isolates of *Candida albicans* resistant to fluconazole. Biol Pharm Bull 31: 2234–2236. 19043205 10.1248/bpb.31.2234

[12] An M, Shen H, Cao Y, Zhang J, Cai Y, et al (2009) Allicin enhances the oxidative damage effect of amphotericin B against *Candida albicans* . Int J Antimicrob Agents 33: 258–263. 19095412 10.1016/j.ijantimicag.2008.09.014

[13] Sharma M, Manoharlal R, Negi AS, Prasad R (2010) Synergistic anticandidal activity of pure polyphenol curcumin I in combination with azoles and polyenes generates reactive oxygen species leading to apoptosis. FEMS Yeast Res 10: 570–578. 20528949 10.1111/j.1567-1364.2010.00637.x

[14] Guo N, Ling G, Liang X, Jin J, Fan J, et al (2011) In vitro synergy of pseudolaric acid B and fluconazole against clinical isolates of *Candida albicans* . Mycoses 54: e400–406. 21910756 10.1111/j.1439-0507.2010.01935.x

[15] Ahmad A, Khan A, Khan LA, Manzoor N (2010) In vitro synergy of eugenol and methyleugenol with fluconazole against clinical *Candida* isolates. J Med Microbiol 59: 1178–1184. 20634332 10.1099/jmm.0.020693-0

[16] Belinky PA, Aviram M, Fuhrman B, Rosenblat M, Vaya J (1998) The antioxidative effects of the isoflavan glabridin on endogenous constituents of LDL during its oxidation. Atherosclerosis 137: 49–61. 9568736 10.1016/s0021-9150(97)00251-7

[17] Carmeli E, Harpaz Y, Kogan NN, Fogelman Y (2008) The effect of an endogenous antioxidant glabridin on oxidized LDL. J Basic Clin Physiol Pharmacol 19: 49–63. 19024795 10.1515/jbcpp.2008.19.1.49

[18] Hasanein P (2011) Glabridin as a major active isoflavan from Glycyrrhiza glabra (licorice) reverses learning and memory deficits in diabetic rats. Acta Physiol Hung 98: 221–230. 21616781 10.1556/APhysiol.98.2011.2.14

[19] Shang H, Cao S, Wang J, Zheng H, Putheti R (2010) Glabridin from Chinese herb licorice inhibits fatigue in mice. Afr J Tradit Complement Altern Med 7: 17–23. 10.4314/ajtcam.v7i1.57225 PMC3005384 21304608

[20] Tsai YM, Yang CJ, Hsu YL, Wu LY, Tsai YC, et al (2011) Glabridin inhibits migration, invasion, and angiogenesis of human non-small cell lung cancer A549 cells by inhibiting the FAK/rho signaling pathway. Integr Cancer Ther 10: 341–349. 21059620 10.1177/1534735410384860

[21] Kwon HS, Oh SM, Kim JK (2008) Glabridin, a functional compound of liquorice, attenuates colonic inflammation in mice with dextran sulphate sodium-induced colitis. Clin Exp Immunol 151: 165–173. 18005263 10.1111/j.1365-2249.2007.03539.x PMC2276918

[22] Hsu YL, Wu LY, Hou MF, Tsai EM, Lee JN, et al (2011) Glabridin, an isoflavan from licorice root, inhibits migration, invasion and angiogenesis of MDA-MB-231 human breast adenocarcinoma cells by inhibiting focal adhesion kinase/Rho signaling pathway. Mol Nutr Food Res 55: 318–327. 20626003 10.1002/mnfr.201000148

[23] Yu XQ, Xue CC, Zhou ZW, Li CG, Du YM, et al (2008) In vitro and in vivo neuroprotective effect and mechanisms of glabridin, a major active isoflavan from Glycyrrhiza glabra (licorice). Life Sci 82: 68–78. 18048062 10.1016/j.lfs.2007.10.019

[24] Fatima A, Gupta VK, Luqman S, Negi AS, Kumar JK, et al (2009) Antifungal activity of Glycyrrhiza glabra extracts and its active constituent glabridin. Phytother Res 23: 1190–1193. 19170157 10.1002/ptr.2726

[25] Messier C, Grenier D (2011) Effect of licorice compounds licochalcone A, glabridin and glycyrrhizic acid on growth and virulence properties of *Candida albicans* . Mycoses 54: e801–806. 21615543 10.1111/j.1439-0507.2011.02028.x

[26] Roling EE, Klepser ME, Wasson A, Lewis RE, Ernst EJ, et al (2002) Antifungal activities of fluconazole, caspofungin (MK0991), and anidulafungin (LY 303366) alone and in combination against *Candida spp*. and *Crytococcus neoformans* via time-kill methods. Diagn Microbiol Infect Dis 43: 13–17. 12052624 10.1016/s0732-8893(02)00361-9

[27] Tanida T, Okamoto T, Ueta E, Yamamoto T, Osaki T (2006) Antimicrobial peptides enhance the candidacidal activity of antifungal drugs by promoting the efflux of ATP from Candida cells. Journal of Antimicrobial Chemotherapy 57: 94–103. 16291868 10.1093/jac/dki402

[28] Di Santo R (2010) Natural products as antifungal agents against clinically relevant pathogens. Nat Prod Rep 27: 1084–1098. 20485730 10.1039/b914961a

[29] Motsei ML, Lindsey KL, van Staden J, Jager AK (2003) Screening of traditionally used South African plants for antifungal activity against *Candida albicans* . J Ethnopharmacol 86: 235–241. 12738093 10.1016/s0378-8741(03)00082-5

[30] Hojo H, Sato J (2002) Antifungal activity of licorice (Glycyrrhiza Glabra) and potential applications to production of beverages. Foods Food Ingredients J Jpn 203: 27–33.

[31] Fu Z, Lu H, Zhu Z, Yan L, Jiang Y, et al (2011) Combination of baicalein and Amphotericin B accelerates *Candida albicans* apoptosis. Biol Pharm Bull 34: 214–218. 21415530 10.1248/bpb.34.214

[32] Sun LM, Cheng AX, Wu XZ, Zhang HJ, Lou HX (2010) Synergistic mechanisms of retigeric acid B and azoles against *Candida albicans* . J Appl Microbiol 108: 341–348. 20002912 10.1111/j.1365-2672.2009.04429.x

[33] Lee JA, Chee HY (2010) In Vitro Antifungal Activity of Equol against *Candida albicans* . Mycobiology 38: 328–330. 23956675 10.4489/MYCO.2010.38.4.328 PMC3741528

[34] da Silva CR, de Andrade Neto JB, de Sousa Campos R, Figueiredo NS, Serpa Sampaio L, et al (2014) Synergistic Effect of the Flavonoid Catechin, Quercetin, or Epigallocatechin Gallate with Fluconazole Induces Apoptosis in *Candida tropicalis* Resistant to Fluconazole. Antimicrobial Agents and Chemotherapy 58: 1468–1478. 24366745 10.1128/AAC.00651-13 PMC3957875

