# Supplementary figures and images for: Synergistic Antifungal Effect of Glabridin and Fluconazole
Source: PLoS One. 2014 Jul 24;9(7):e103442. doi: 10.1371/journal.pone.0103442 (PMC4110026; doi:10.1371/journal.pone.0103442)

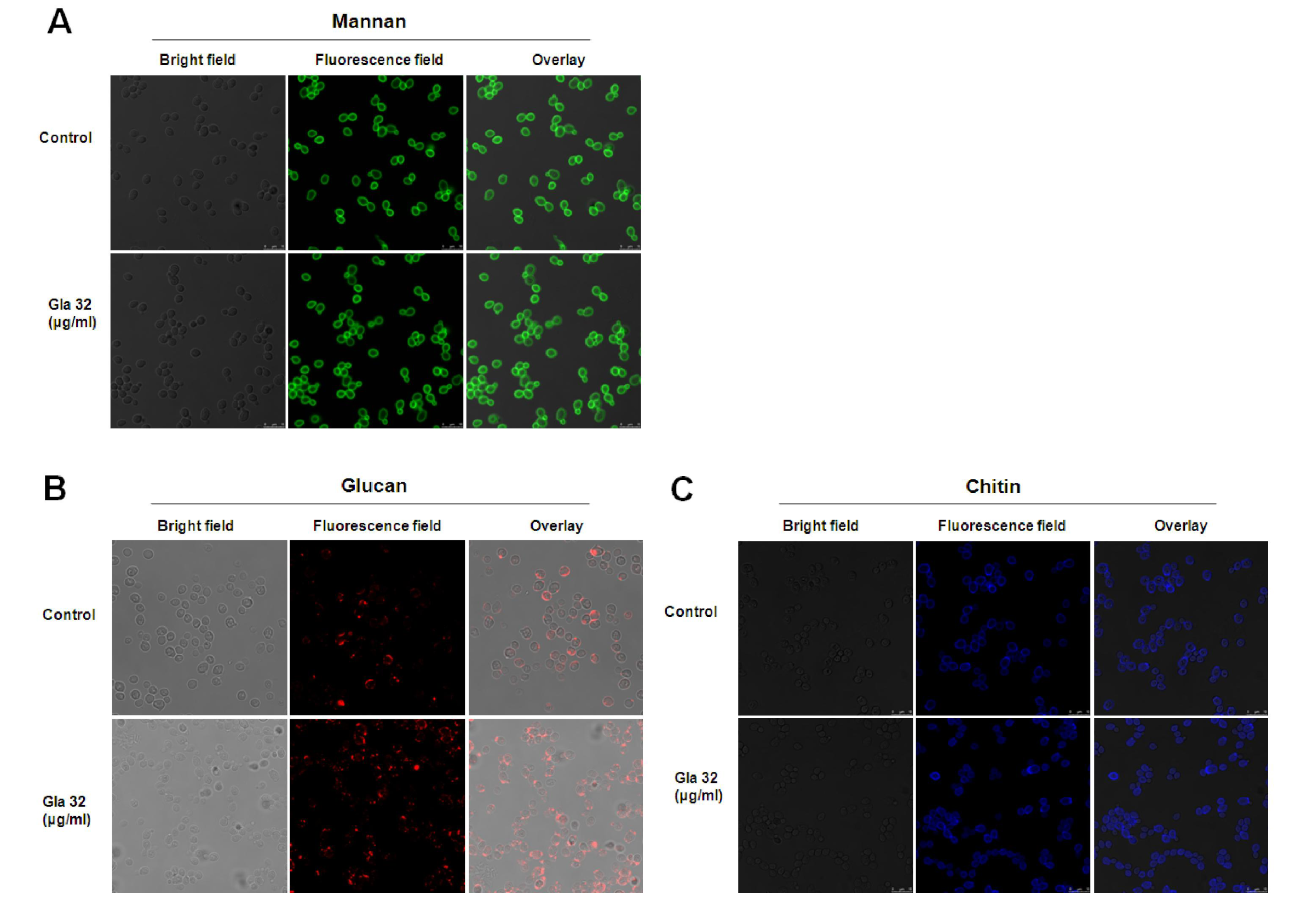

Supplement: Figure S1 — Fluorescence micrographs of the cell wall structures of Candida albicans by the treatment of Gla. Exponentially growing cells treated with or without 32 µg/ml Gla were stained by 50 µg/ml Concanavalin A alexa fluor 488 conjugate for mannan, 30 µg/ml Calcofluorwhite for chitin, or specific anti-β-glucan primary antibody and Cy3-labeled goat-anti mouse secondary antibody for glucan. Then cells were scanned under a Leica confocal laser scanning microscope and micrographs were acquired. (TIF) [file pone.0103442.s001.tif]
